# Supplementary material for: Decline in male circumcision in South Korea
Source: BMC Public Health. 2012 Dec 11;12:1067. doi: 10.1186/1471-2458-12-1067 (PMC3526493; doi:10.1186/1471-2458-12-1067)
Supplement: Additional file 1 — The questionnaire used in our survey; the questions were simplified to draw maximum response rate. [file 1471-2458-12-1067-S1.pdf]

Questionnaire (Self)

1. How old are you?<br>2. Are you circumcised?
3. If you are circumcised, how old were you when you were circumcised?<br>4. Have you been in contact with information regarding circumcision; or Have you look for information regarding circumcision?<br>5. If you have been in contact with information regarding circumcision, has it primarily been:<br>① pro-circumcision<br>② anti-circumcision<br>③ both sides<br>6. Where did you find information on circumcision? (multiple choices allowed)<br>① internet ② newspaper ③ book ④ lecture ⑤ television ⑥ other

Questionnaire (Parent)

1. How old are you?<br>2. How old is your son?<br>3. Is he circumcised?
4. If he circumcised, how old was he when he was circumcised?<br>5. Have you been in contact with information regarding circumcision; or Have you look for information regarding circumcision?<br>6. If you have been in contact with information regarding circumcision, has it primarily been:<br>① pro-circumcision<br>② anti-circumcision<br>③ both sides<br>7. Where did you find information on circumcision? (multiple choices allowed)<br>① internet ② newspaper ③ book ④ lecture ⑤ television ⑥ other
